# Ghrelin Indirectly Activates Hypophysiotropic CRF Neurons in Rodents

**DOI:** 10.1371/journal.pone.0031462

**Published:** 2012-02-20

**Authors:** Agustina Cabral, Olga Suescun, Jeffrey M. Zigman, Mario Perello

**Affiliations:** 1 Laboratory of Neurophysiology, Multidisciplinary Institute of Cell Biology, Argentine Research Council (CONICET) and Scientific Research Commission, Province of Buenos Aires (CIC-PBA), La Plata, Buenos Aires, Argentina; 2 Laboratory of Reproductive Endocrinology, Multidisciplinary Institute of Cell Biology, Argentine Research Council (CONICET) and Scientific Research Commission, Province of Buenos Aires (CIC-PBA), La Plata, Buenos Aires, Argentina; 3 Divisions of Hypothalamic Research and Endocrinology and Metabolism, Department of Medicine, The University of Texas Southwestern Medical Center, Dallas, Texas, United States of America; 4 Department of Psychiatry, The University of Texas Southwestern Medical Center, Dallas, Texas, United States of America; University of Michigan School of Medicine, United States of America

## Abstract

Ghrelin is a stomach-derived hormone that regulates food intake and neuroendocrine function by acting on its receptor, GHSR (*Growth Hormone Secretagogue Receptor*). Recent evidence indicates that a key function of ghrelin is to signal stress to the brain. It has been suggested that one of the potential stress-related ghrelin targets is the CRF (*Corticotropin-Releasing Factor*)-producing neurons of the hypothalamic paraventricular nucleus, which secrete the CRF neuropeptide into the median eminence and activate the hypothalamic-pituitary-adrenal axis. However, the neural circuits that mediate the ghrelin-induced activation of this neuroendocrine axis are mostly uncharacterized. In the current study, we characterized *in vivo* the mechanism by which ghrelin activates the hypophysiotropic CRF neurons in mice. We found that peripheral or intra-cerebro-ventricular administration of ghrelin strongly activates c-fos – a marker of cellular activation – in CRF-producing neurons. Also, ghrelin activates CRF gene expression in the paraventricular nucleus of the hypothalamus and the hypothalamic-pituitary-adrenal axis at peripheral level. Ghrelin administration directly into the paraventricular nucleus of the hypothalamus also induces c-fos within the CRF-producing neurons and the hypothalamic-pituitary-adrenal axis, without any significant effect on the food intake. Interestingly, dual-label immunohistochemical analysis and ghrelin binding studies failed to show GHSR expression in CRF neurons. Thus, we conclude that ghrelin activates hypophysiotropic CRF neurons, albeit indirectly.

## Introduction

Ghrelin is a peptide hormone derived from the gastro-intestinal tract [1]. Ghrelin acts on the central nervous system to regulate different actions, including the control of growth hormone secretion, food intake/energy expenditure regulation and glucose homeostasis regulation [1]–[4]. Recent evidence indicates that ghrelin also participates in physiological responses to various forms of stress [5]. We and others have found that plasma ghrelin rises in response to stress induced by acute or chronic caloric restriction [6]–[10]. Ghrelin also rises in response to various forms of acute or chronic psychological stress. For instance, elevations in gastric ghrelin gene expression and plasma ghrelin have been also observed in rodents' response to acute stress, including tail pinch stress and water avoidance stress [11], [12]. Rises in plasma ghrelin levels were shown in rodents stressed by chronic social defeat or exposure to a continuously flooded cage [13]–[15]. Importantly, human beings subjected acutely to psychosocial stress also display increased plasma ghrelin [16]. Mice with genetic deletion of GHSR were unable to respond as wild-type mice to stress-induced alterations of mood, feeding and metabolism suggesting that elevated plasma ghrelin participates in stress-associated responses [5], [13], [15]. However, the circuit by which ghrelin engages these responses are not yet elucidated.

One potential mechanism might include activation of the hypothalamic-pituitary-adrenal (HPA) neuroendocrine axis, which is one of the targets of ghrelin's central actions. In response to stress, activation of corticotropin-releasing factor (CRF)-producing neurons of the paraventricular nucleus of the hypothalamus (PVN) leads to secretion of CRF into the median eminence, and a subsequent stimulation of adrenocorticotropic hormone (ACTH) secretion from the pituitary gland [17]. In turn, ACTH acts on the adrenal cortex to release glucocorticoids that have a variety of important effects including increases in glycemia and body weight [18]. Recently, a role of ghrelin as an activator of the HPA axis has been documented [19], [20]. Ghrelin appears to activate the HPA axis by acting at the hypothalamic level. For instance, ghrelin releases CRF from hypothalamic explants *in vitro* [19], [20]. Also, centrally administrated ghrelin stimulates CRF mRNA expression in the hypothalamus and increases ACTH and glucocorticoid plasma levels in rodents [19], [21]. Importantly, ghrelin administration strongly stimulates ACTH and cortisol release in healthy lean humans [22], [23]. It is yet unclear, however, whether ghrelin's effects on the HPA axis occur via direct regulation of CRF-producing neurons or via indirect pathways.

In the present study, we systematically examined the responses of CRF-producing neurons and of the HPA axis in mice after peripheral and central ghrelin treatment. To further assess the functional consequence of ghrelin actions on the PVN, we directly administered the hormone into this nucleus. Finally, we assessed if CRF-producing neurons express ghrelin receptor. Our results indicate that peripheral, central and intra-PVN ghrelin administration strongly activate hypophysiotropic CRF neurons and, as a consequence, the HPA axis. This action, however, seems to be indirect since CRF neurons fail to express GHSR.

## Materials and Methods

### Animals

Adult (2–3 months old) C57BL6/J mice were from Jackson Laboratory or generated in the animal facility of the IMBICE. Male mice were housed in a 12-h light/dark cycle with regular chow and water available *ad lib*. All experimentation received approval from the Institutional Animal Care and Use Committees located in The University of Texas Southwestern Medical Center (approval ID 1090-06-06-1) and Multidisciplinary Institute of Cell Biology (approval ID 10-0112).

### Treatments and stereotaxic surgeries

In the first experiment, mice were subcutaneously injected with saline or acyl-ghrelin (2 µg/g body weight, Global Peptide, cat. #C-et-004), as previously done [9]. For central infusion of ghrelin, two different variants of stereotaxic surgeries were performed. For one experiment, mice were stereotaxically implanted with a single indwelling sterile guide cannula (4 mm long, 22 gauge, Plastics One, Inc. Roanoke, VA) into the lateral ventricle (intra-cerebro-ventricular, ICV). The placement coordinates for the lateral ventricle were: AP:−0.34 mm; L:+1 mm and V:−2.3 mm. A 28-gauge obturator was inserted into each cannula. After surgery, animals were individually housed and allowed to recover for at least 5 days. Mice were made accustomed to handling by removal of the dummy cannula and connection to an empty cannula connector daily for at least 4 days prior to experimentation, to reduce stress. Correct placement of the cannula was confirmed by histological observation at the end of the experiment. On the morning of the experimental day, animals were ICV injected with 4 µL of vehicle (artificial cerebrospinal fluid, aCSF) with or without acyl-ghrelin (2 µg/mouse). We have successfully studied the actions of ghrelin on food intake using this protocol, previously [24]. All ICV injections were made over 2 min through a 30 gauge needle that extend 0.5 mm below the guide cannula and that was connected by polyethylene tubing to a 5 µL Hamilton syringe. The needle was left in place for 2 min, following the injection, to prevent back flow of the injected solution. Between injections and sacrifices, mice were exposed to a pre-weighed amount of rodent's food, except when indicated. In another experiment, mice were stereotaxically implanted with a double indwelling sterile guide cannula (10 mm long, 26 gauge, Plastics One, Inc. Roanoke, VA). The placement coordinates were: AP:−0.82 mm; L:±0.5 mm and V:−2.0 mm. After surgery, mice were manipulated as described above. On the morning of the experimental day, animals were injected with a 30 gauge needle that was extended to 2.8 mm below the guide cannula; thus, allowing injections to be made 500 µm dorsal to the PVN. We administered 0.5 µL of sterile saline with or without acyl-ghrelin (0.1 µg) on each side of the PVN. Injections were made with a 2 µL Hamilton syringe, over 2 min. PVN injections were verified post-mortem by histological visualization of the injection cannula tracts. Finally, one group of mice was stereotaxically injected with colchicine (16 µg in 4 µL per mouse, Sigma-Aldrich) into the lateral ventricle using the above described coordinates. After surgery, animals were allowed to recover for 2 days and used as described below.

### Assessment of c-fos and CRF colocalization by immunohistochemistry (IHC)

Two hours after vehicle or ghrelin treatment, anesthetized mice were perfused with formalin as previously described [10]. This time point was chosen based on previous evidence showing that increases of c-fos gene expression occur after CRF gene expression and that the presence of nuclear c-fos protein requires not only gene expression, but also protein biosynthesis and mobilization from cytoplasm to the cell nucleus [25], [26]. Brains were removed, post-fixed, immersed in 20% sucrose, and cut coronally at 20 µm into three equal series on a sliding cryostat. Double c-fos/CRF staining was performed as described [9]. Briefly, sections were pretreated with 1% H_2_O_2_, treated with blocking solution (3% normal donkey serum and 0.25% TritonX in PBS), and incubated with anti-c-fos antibody (Calbiochem/Oncogene, Temecula, CA, cat# PC38, 1∶15,000) for two days at 4°C. Then, sections were treated with biotinylated donkey anti-rabbit antibody (Jackson ImmunoResearch Laboratories, West Grove, PA, 1∶1,000) for 1 h, and with Vectastain Elite ABC kit (Vector Laboratories, Burlingame, CA) for 1 h, according to manufacturer's protocols. Then, visible signal was developed with 3-3′-diaminobenzidine (DAB)/Nickel solution, giving a black/purple precipitate. Consecutively, sections were washed and incubated overnight with anti-CRF antibody (1∶2,000). This antibody recognizes mature CRF(1–41) and full pre-proCRF prohormone [27]. The next day, sections were sequentially incubated with biotinylated donkey anti-rabbit antibody and Vectastain Elite ABC kit as detailed above. Finally, visible signal was developed by incubation with DAB solution, giving a brown precipitate. Sections were sequentially mounted on glass slides, and cover slipped with mounting media. Results were visualized using bright-field light sources. Bright-field images were acquired with a Nikon Eclipse 50i (Nikon, Japan) and a DS-Ri1 Nikon digital camera (Nikon, Japan). An image editing software program, Adobe Photoshop CS2 (San Jose, CA), was used to adjust contrast and brightness. CRF immunostaining was confined to the perikarya and dendrites, thus allowing visualization of the nucleus with or without black/purple label for c-fos. The relationship was expressed as a percentage, which represents CRF immunoreactive (IR) neurons positive for c-fos compared to the total number of CRF-IR neurons observed. The data were corrected for double counting, according to the method of Abercrombie [28] where the ratio of the actual number of neurons to the observed number is represented by T/T+h where T = section thickness, and h = the mean diameter of the neuron along the axis perpendicular to the plane of section. The mean diameter of the neurons was determined with the software program Image J. For the analysis, the mouse PVN was subdivided into five parts, which were named: compact, periventricular, anterior, medial and posterior, as previously suggested [29]. The percentage of CRF neurons containing labeled nuclei was determined in the compact part of the PVN, located between −0.70 mm and −0.94 mm from bregma, where CRF neurons are enriched and most hypophysiotropic PVN neurons are located (see below) [30]. Quantitative analysis was performed in 3–4 animals per condition.

### Assessment of responsiveness of the HPA axis to ghrelin

ICV ghrelin- or vehicle-treated animals were sacrificed by decapitation at 0, 15, 30, 60 or 120 min after treatment (n = 5−6 per group per time point). Added food was weighed as was food remaining at the end of each time point, and food intake was calculated by subtracting these values from one another. Blood samples were immediately collected and processed for corticosterone analysis, which was accomplished using an ELISA kit according to the procedures and reagents provided (Assay Designs). Brains were extracted, placed in cold diethylpyrocarbonate-phosphate buffered saline (PBS), and sectioned into 1 mm coronal slices by use of a mouse brain matrix. Small “punches” of tissue corresponding to the location of PVN (identified by comparing the coronal slices to a mouse brain atlas) were excised using a 15 g needle. Cannula position was verified at the end of the experiment by visualization of the injection cannula tracts. PVN punches were collected in TRIzol Reagent (Invitrogen Inc, Carlsbad, CA).Total RNA was isolated from PVN punches using RNA STAT-60 (Tel-Test Inc.) and quantified by absorbance at 260 nm. The total RNA was treated with RNase-free DNase (Roche) and reverse-transcribed into cDNA with random hexamer primers and SuperScript II reagents (Invitrogen). Quantitative PCR was performed using an Applied Biosystems 7900HT sequence detection system and SYBR-green chemistry (Applied Biosystems, Foster City, CA). The CRF mRNA levels are expressed relative to the housekeeping gene Cyclophilin A, calculated by the comparative threshold cycle (Ct) method, and then the data is presented as a percentage of levels observed in PVN punches. Primers sequences for CRF were Sense: 5′-TCTGGATCTCACCTTCCACCT-3′, Antisense: 5′-CCATCAGTTTCCTGTTGCTGT-3′ [GenBank Accession No. NM_205769], product size 95. Primers sequences for Cyclophilin A Sense: 5′-TGGTCTTTGGGAAGGTGAAAG-3′, Antisense: 5′-TGTCCACAGTCGGAAATGGT-3′ [GenBank Accession No. NM_008907] product size 109. All reactions were performed in triplicate in sealed fast optical 96-well reaction plates (Applied Biosystems). Standard curves for CRF and Cyclophilin A transcript levels were generated using hypothalamic cDNA of mouse with ABI 7500 Fast System SDS Software version 1.3.1 (Applied Biosystems). Averaged levels of CRF normalized to Cyclophilin A in each experimental group were compared with similar values obtained from vehicle-treated mice to determine relative expression levels.

### Visualization of ghrelin binding sites in brain sections

The procedure for ghrelin binding was adapted from a previously-described protocol [31]. Initially, brain sections were pretreated with 0.5% H_2_O_2_ and treated with blocking solution (3% normal donkey serum and 0.25% TritonX in PBS). Then, brain sections were incubated with acyl-ghrelin (10 µg/mL, Global Peptide, cat #C-et-004) in PBS for 1 hour at 37°C. Next, coronal sections were brought to room temperature, dipped in 0.5% formaldehyde for 30 minutes, and then rinsed six times in PBS buffer. Then, brain sections were incubated with goat anti-ghrelin antibody (Santa Cruz, cat# sc-10368, 20 µg/mL) overnight at room temperature. The next day, sections were washed and treated with biotinylated donkey anti-goat antibody (Jackson ImmunoResearch Laboratories, West Grove, PA, 1∶1,000) for 1 h, and with Vectastain Elite ABC kit (Vector Laboratories, Burlingame, CA) for 1 h, according to manufacturer's protocols. Finally, visible signal was developed with DAB/Nickel solution, giving a black/purple precipitate. To determine the specificity of the reaction, coronal sections were incubated with decreasing concentrations of ghrelin (0–1 µg/mL) and the intensity of signal was subsequently measured. Negative controls were also performed using the same procedure but omitting the primary antibody. Sections were sequentially mounted on glass slides, and cover slipped with mounting media. Results were visualized using bright-field light sources. Bright-field images were acquired with a Nikon Eclipse 50i (Nikon, Japan) and a DS-Ri1 Nikon digital camera (Nikon, Japan).

### Assesment of GHSR mRNA levels in the PVN and ARC

Anesthetized mice were sacrificed by decapitation. PVN and ARC punches from these mice were used to isolate total RNA, which was then submitted to reverse-transcription into cDNA and quantitative PCR, as described above. Primers mGHSR-QF1, 5′-ACCGTGATGGTATGGGTGTCG-3′ and mGHSR-QR1, 5′-CACAGTGAGGCAGAAGACCG-3′ amplify a product within Exon 2 of the GHSR gene. These primers were previously extensively validated using template titration and dissociation curves [15].

### Assessment of dual GHSR mRNA expression and CRF immunoreactivity in the PVN

This was performed by using *in situ* hybridization histochemistry (ISHH) for GHSR and IHC for CRF. Two days after colchicine treatment, animals were perfused and brain slice sections obtained as described above. Free-floating sections of mouse brains were processed sequentially by ISHH and then IHC using a protocol reported previously [10]. Series of three different mouse brains were processed for GHSR-CRF co-expression. Briefly, brain sections were thoroughly washed and incubated with a^35^S-labeled GHSR mouse cRNA riboprobe [10^6^ cpm/mL in a hybridization solution previously reported], which was generated as previously explained in detail [10]. Next, sections were incubated at 57°C overnight in hybridization solution. Subsequently, sections were rinsed, treated with RNase solution A (Roche Molecular Biochemicals, Indianapolis, IN) and thoroughly washed with stringent buffers. IHC was begun on the same ISHH-processed sections. Sections were treated as explained above and incubated overnight at room temperature in polyclonal anti-CRF antiserum made in goat (Santa Cruz. no. A-6455, lot 71B1, 1∶2,000 in PBT-azide). Next day, section were developed with DAB as described above and mounted onto SuperFrost Plus slides (Fisher Scientific, Pittsburgh, PA). ISHH patterns were visualized first on autoradiographic film and then by observing slides dipped in photographic emulsion for direct, cellular visualization. Of note, one series of coronal sections from each mouse was used for ISHH plus thionin-counterstaining, without any further IHC staining. Brain sections were viewed with both a Zeiss Axioskop and a Zeiss Stemi 2000-C dissecting microscope using both brightfield and darkfield optics. Photomicrographs were produced with a Zeiss digital camera. Criteria used to determine whether a CRF-IR cell co-expressed GHSR mRNA included both 1) bright field visualization of silver granules overlying the DAB-stained cell at 5× the background density of silver granule deposition, and 2) conformation of the overlying silver granules to the shape of the DAB-stained cell. Cell counts were performed on every third section of each mouse brain, as done in the past [10]. Estimates of cell counts were performed using a 10× objective. An image editing software program, Adobe PhotoShop 7.0 (San Jose, CA), was used to adjust sharpness, contrast and brightness in the photomicrographs.

### Statistical analyses

Data is expressed as the mean±SEM. The t-test for each time point was performed when assessing ghrelin-induced changes on CRF gene expression and plasma corticosterone. One-way ANOVA was used to compare percentage CRF-IR neurons positive for c-fos in the compact part of the PVN since there were three experimental groups per experiment (vehicle, ghrelin/food and ghrelin/no food). Also, one-way ANOVA was performed when comparing CRF gene expression and plasma corticosterone data of ghrelin microinjection into the PVN. Significant differences were considered when P<0.05.

## Results

### Peripheral ghrelin administration activates CRF neurons of the PVN and the HPA

**Figure 1:**
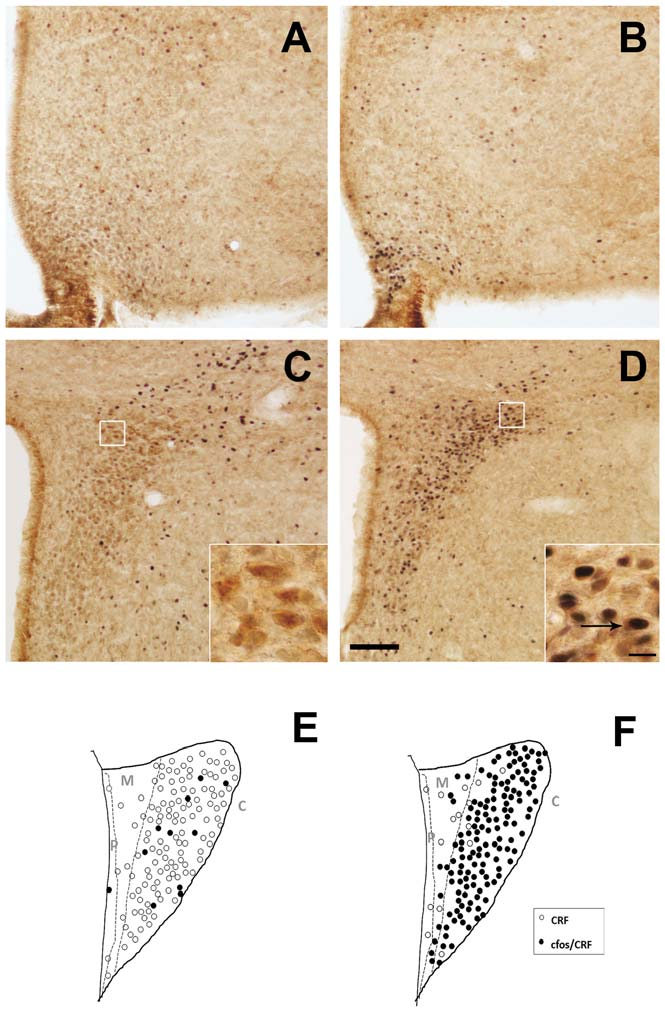
Ghrelin administration activates CRF neurons of the PVN. Panels **A** to **D** show representative photomicrographs of brains sections subjected to double immunohistochemistry using anti-CRF (brown staining) and anti-c-fos (purple-black staining) antiserum. Upper panels show the ARC and bottom panels show the PVN. Images of panel **A** and **C** are from a vehicle-treated mouse and images of panel **B** and **D** from a ghrelin-treated mouse. Inserts in PVN images show high magnification of areas marked in low magnification images. Arrows point to dual-labeled cells. Scale bars, 100 µm (low magnification), 10 µm (high magnification). Panels **E** and **F** show schematic drawings of middle level of the PVN of a vehicle- and ghrelin-treated mouse, respectively. Filled circles represent CRF-IR neurons positive for c-fos, and open circles indicate single-labeled CRF-IR cell bodies. Gray letters indicate the PVN subdivisions: P: periventricular; M: medial; C: compact.

To ascertain whether ghrelin modulates CRF neurons and the HPA axis, we injected *ad lib*-fed C57BL6/J mice with ghrelin (2 µg/g BW, sc) or saline. In our experimental conditions, we detected 2962±276 CRF-IR neurons within the combined mouse PVNs, with an average cytoplasmic diameter of 10.5±0.6 µm. CRF-IR neurons were located throughout the whole rostral-to-caudal axis of the PVN, although they were strongly enriched within the compact part in the middle level of the nucleus. Within this compact part, 67.4±1.8% of all CRF-IR PVN neurons were concentrated. We found that ghrelin, 2 h after administration, induces a profound increase of c-fos immunoreactivity within both the arcuate (ARC, **Figure 1A–B**) and the PVN (**Figure 1C–D**). The total number of CRF-IR cells in the PVN (2714±217 neurons) and their average diameter (10.3±0.5 µm) were not significantly affected by acute subcutaneous ghrelin administration. In the PVN, ghrelin-treated mice had a significant increase in the percentage of CRF-IR neurons positive for c-fos as compared to the levels found in vehicle-treated animals. Double positive cells were particularly observed in compact part of the PVN in ghrelin-treated mice (**Figure 1E–F**). In the compact part, the quantitative analysis indicated that 12.1±6.6% of the CRF-IR cells were positive for c-fos in control animals. Conversely, 72.9±5.4% of CRF-IR neurons were positive for c-fos in ghrelin-treated animals (p<0.01 vs. vehicle-treated group, n = 3 per group). Importantly, ghrelin-treated mice with no access to food had 64.1±6.3% of CRF-IR neurons positive for c-fos in the compact part of the PVN (p<0.01 vs. vehicle-treated group, n = 3 per group), suggesting that the ghrelin-induced activation CRF neurons of the PVN is independent of the ghrelin-induced increase of food intake. The responsiveness of the HPA axis was quantified by measurements of plasma glucocorticoids. Peripheral administration of ghrelin significantly increased plasma corticosterone levels one hour after treatment to 10.3±2.4 ng/mL, as compared to vehicle-treated mice that had 2.1±0.4 ng/mL (p<0.01, n = 7 per group). Plasma corticosterone levels also increased, one hour after treatment, in ghrelin-treated mice that had no access to food (11.4±1.9 ng/mL, n = 4).

### Central ghrelin administration activates CRF neurons of the PVN and the HPA axis

**Figure 2:**
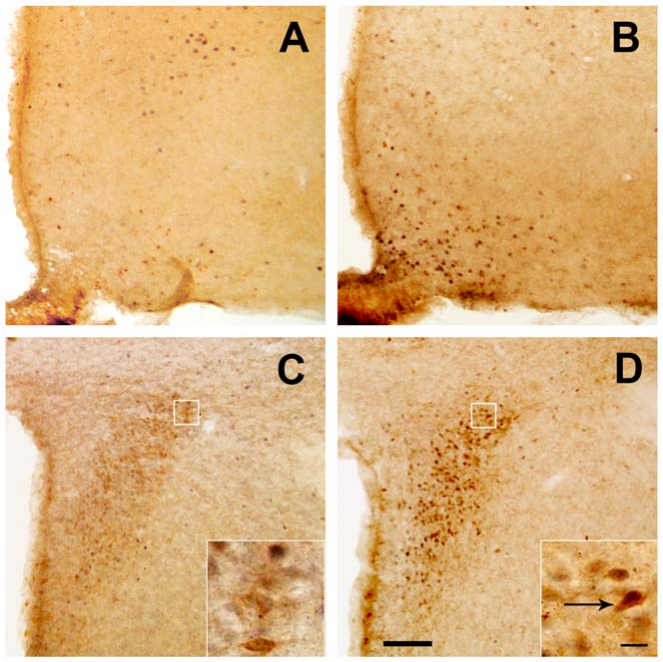
ICV ghrelin administration activates CRF neurons of the PVN. Panels show representative photomicrographs of brains sections subjected to double immunohistochemistry using anti-CRF (brown staining) and anti-c-fos (purple-black staining) antiserum. Upper panels show the ARC and bottom panels show the PVN. Images of panel **A** and **C** are from a vehicle-treated mouse and images of panel **B** and **D** from a ghrelin-treated mouse. Inserts in PVN images show high magnification of areas marked in low magnification images. Arrows point to dual-labeled cells. Scale bars, 100 µm (low magnification), 10 µm (high magnification).

In order to rule out any potential contribution of peripheral actions of ghrelin to these observations, we tested the response of CRF PVN neurons and the HPA axis to central administration of the hormone. In this experiment, we injected *ad lib*-fed mice with ghrelin (2 µg in 4 µL per mouse, ICV) or vehicle. Central ghrelin administration increased c-fos immunoreactivity in the ARC (**Figure 2A–B**). In the compact part of the PVN, we observed a significant increase of the percentage of CRF-IR neurons positive for c-fos, 2 h after treatment (**Figure 2C–D**). In this part, 10.4±1.6% of the CRF-IR cells were positive for c-fos in vehicle-treated animals, while 72.2±11.1% of CRF-IR neurons were positive for c-fos in ghrelin-treated mice (p<0.01 vs. vehicle-treated group, n = 3 per group). The total number or cytoplasmic diameter of CRF-IR cells in the PVN was not affected by ICV ghrelin (data not shown). The percentage of CRF-IR neurons positive for c-fos in the PVN also increased in ICV ghrelin-treated mice that had no access to food (87.6±4.6%, p<0.01 vs. vehicle-treated group, n = 3). Thus, the ghrelin-induced activation of CRF neurons of the PVN seems to be independent of access to ghrelin-induced increase of food intake, as observed with peripheral administration of ghrelin.

**Figure 3:**
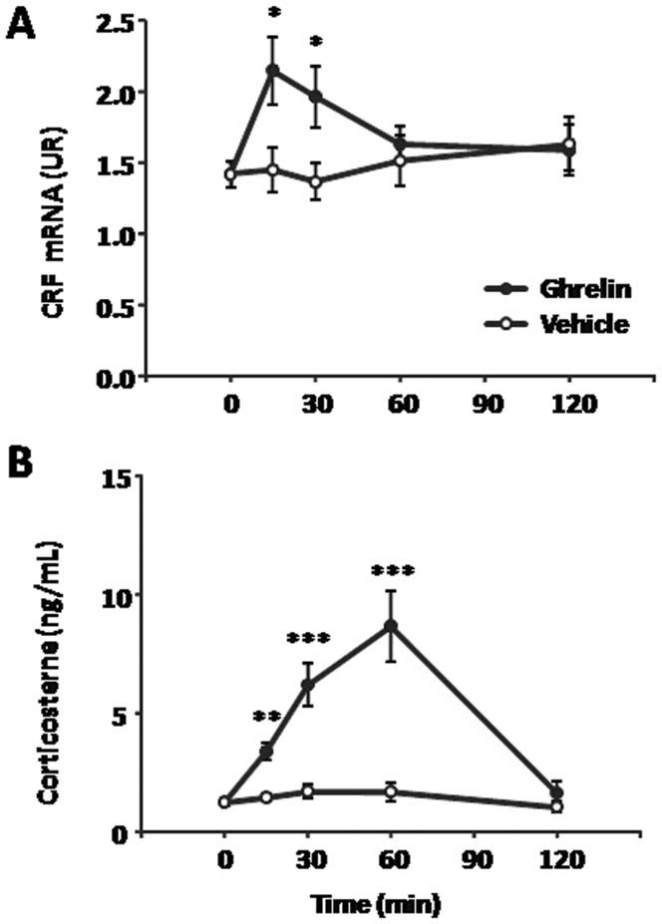
ICV ghrelin administration activates the HPA axis. Panel **A** shows comparative values of RT-qPCR for CRF mRNA in the PVN micro-dissected punches obtained from vehicle-treated and ghrelin-treated mice sacrificed at different time points. Data is shown as mRNA levels of CRF relative to the housekeeping gene Cyclophilin A, calculated by the comparative threshold cycle (Ct) method. Panel **B** shows comparative values of specific ELISA for plasma corticosterone obtained from vehicle- and ghrelin-treated mice sacrificed at different time points. Data represent the mean±SEM. *p≤0.05. **p≤0.01. ***p≤0.001.

To further study the role of ghrelin on CRF neurons and more specifically determine the HPA axis response, we sacrificed animals at multiple time points following central ghrelin administration and analyzed changes in CRF gene expression and corticosterone levels. Ghrelin induced a significant increase of CRF gene expression in the PVN at 15 and 30 min post treatment (**Figure 3A**), which went from 1.42±0.09 relative CRF mRNA levels in the control to 2.15±0.24 relative CRF mRNA levels, at 15 min post treatment (P<0.05, n = 5 per group). Also, ghrelin induced a significant increase in plasma corticosterone levels at 15, 30 and 60 min post treatment. The maximum response was observed at 60 min post treatment (**Figure 3B**), when plasma corticosterone was 8.7±1.5 ng/mL in ghrelin-treated mice and 1.7±0.4 ng/mL in saline-treated mice (P<0.05, n = 5 per group).

### Ghrelin microinjection into the PVN activates CRF neurons and the HPA axis

**Figure 4:**
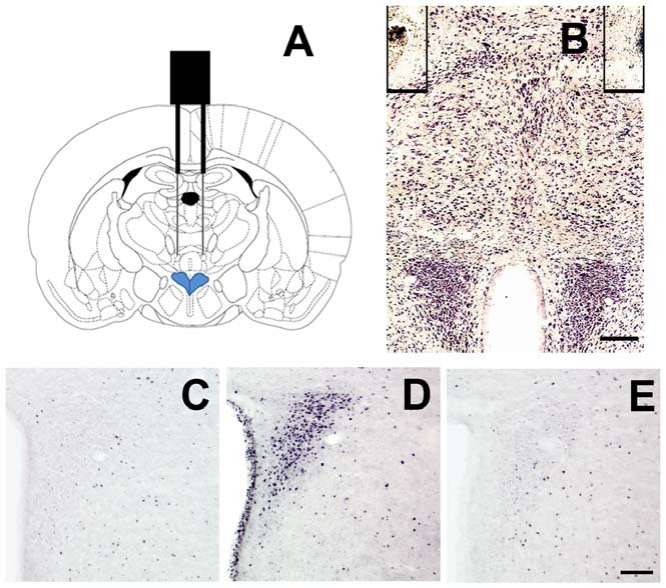
Ghrelin can be microinjected exclusively into the PVN. Panel **A** shows a schematic diagram of a coronal section of the mouse brain with the localization of a correctly implanted cannula (black). This experimental strategy allowed us to administer vehicle, containing or not ghrelin, 500 µm above the PVN (blue) via the injector cannula (gray). Panel **B** shows a representative photomicrograph of a coronal brain section subjected to thionin staining of an intra-PVN injected mouse. Lines label injector cannula tracts, where no thionin staining is observed. Panels **C**, **D** and **E** show representative photomicrographs of brain sections, containing the PVN, subjected to immunohistochemistry using anti-c-fos (purple-black staining) antiserum in “saline-treated”, “ghrelin-hits” and “ghrelin-misses” groups, respectively. Scale bars, 100 µm.

**Figure 5:**
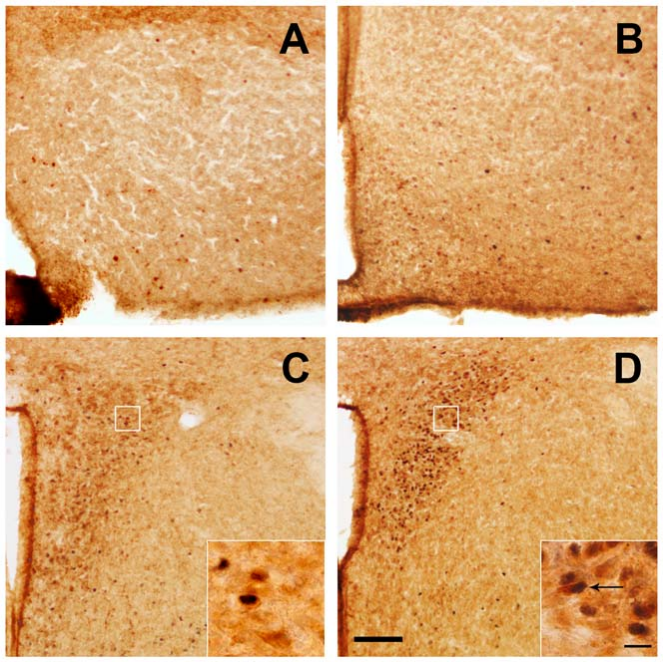
Ghrelin microinjection into the PVN activates CRF neurons. Panels **A** to **B** show representative photomicrographs of brains sections subjected to double immunohistochemistry using anti-CRF (brown staining) and anti-c-fos (purple-black staining) antiserum. Upper panels show the ARC and bottom panels show the PVN. Images of panel **A** and **C** are from a vehicle-treated mouse and images of panel **B** and **D** from a ghrelin-treated mouse. Inserts in **C** and **D** panels show high magnification of areas marked in low magnification images. Arrows to dual-labeled cells. Scale bars, 100 µm (low magnification), 10 µm (high magnification).

**Figure 6:**
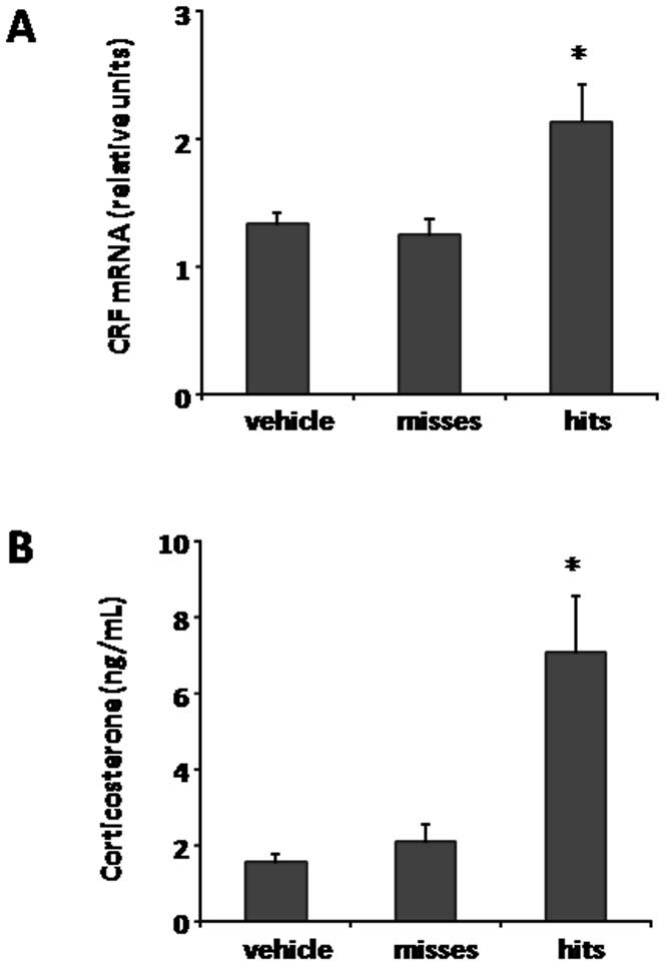
Ghrelin microinjection into the PVN activates the HPA axis. Panel **A** shows comparative values of RT-qPCR for CRF mRNA in the PVN micro-dissected punches obtained from intra-PVN ghrelin-treated, intra-PVN vehicle-treated and misses groups. Data is shown as mRNA levels of CRF relative to the housekeeping gene Cyclophilin A, calculated by the comparative threshold cycle (Ct) method. Panel **B** shows comparative values of specific ELISA for plasma corticosterone obtained from intra-PVN ghrelin-treated, intra-PVN vehicle-treated and misses groups. Data represent the mean±SEM. *p≤0.05.

Ghrelin receptors are expressed in several brain nuclei, including the PVN [32]. In order to focus our studies on the PVN, we micro-injected ghrelin directly to this nucleus via an injector cannula located 500 µm dorsal of each PVN (**Figure 4A–B**). For these studies, we injected *ad lib*-fed mice with ghrelin (0.1 µg in 0.5 µL per side per mouse) or vehicle. Those mice with correctly placed cannulas were analyzed as “hits”, while those mice with incorrectly placed cannulas were separately grouped as “misses”. Two hours after treatment, ghrelin infusion into the PVN produced a massive increase in c-fos immunoreactivity exclusively in this nucleus in the ghrelin-treated “hits” group (**Figure 4D**), as compared to the “saline-treated” group (**Figure 4C**). The ghrelin-treated “misses” group failed to show any significant increase of c-fos signaling within the PVN (**Figure 4E**). In contrast, no c-fos was observed in the ARC of ghrelin-injected mice suggesting that ghrelin's infusion was limited to the PVN, as intended (**Figure 5A–B**). Ghrelin infusion into the PVN increased the percentage of CRF-IR neurons positive for c-fos to 66.8±3.0% in the compact part (**Figure 5D**, n = 3 per group). In contrast, few of the CRF-IR cells were positive for c-fos in the saline-treated group (**Figure 5C**, 17.0±0.5%, p<0.01 vs. ghrelin-treated group, n = 3). Despite this effect on c-fos induction, ghrelin infusion into the PVN did not induce any significant food intake in mice as compared to saline-treated mice and ghrelin-treated “misses”. Ghrelin infused into the PVN induced a significant increase of the relative CRF mRNA levels at 30 min post treatment in ghrelin-treated “hits” group (2.13±0.29) as compared to vehicle-treated mice (1.32±0.10) and “misses” (1.24±0.13) (P<0.05, **Figure 6A**). Also, plasma corticosterone levels significantly increased in ghrelin-treated “hits” (7.1±1.5 ng/mL) at 30 min post treatment as compared to vehicle-treated mice (1.5±0.2 ng/mL) and “misses” (2.1±0.5 ng/mL) (P<0.05, **Figure 6B**).

### CRF-IR neurons of the PVN do not express GHSR

**Figure 7:**
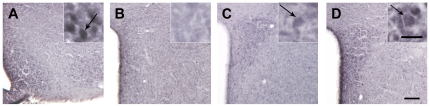
Ghrelin binding to the compact part of the PVN is scarce. This figure shows representative photomicrographs of brains sections subjected to a ghrelin binding assay (see Material and Methods for details), at low and high magnification (inserts). Purple/Black signal indicates specific binding of ghrelin. Panel **A** shows an internal positive control of the assay in which positive ghrelin binding to cells located in the ARC and to the ventrolateral subdivision of the VMH (vlVMH) is observed, when brain section are incubated with 1 µg/ml of ghrelin. Panel **B** shows the PVN level for a negative control, where no exogenous ghrelin was added. Panels **C** and **D** show positive ghrelin binding to cells located in the PVN, in brain section previously incubated with 0.1 or 1 µg/ml of ghrelin, respectively. Inserts in each image show high magnification of areas marked in low magnification images. Arrows point to labeled cells. Scale bars, 100 µm (low magnification), 10 µm (high magnification).

Finally, we tested the capacity of CRF neurons to respond directly to ghrelin by assessing the capacity of exogenous ghrelin to bind to the compact part of the PVN. Internal positive controls for the assay were the ARC and ventromedial hypothalamic nucleus (VMH), where GHSR is expressed [32]. As expected, ghrelin binding was strongly located in cell bodies within the ARC and the ventrolateral subdivision and the capsule of the VMH (**Figure 7A**), as previously described for mouse GHSR expression [32]. The binding assay showed specificity as no signal was observed when ghrelin was omitted from the assay (**Figure 7B**) and the intensity of signal increased when coronal sections were incubated with increasing concentrations of ghrelin (0.1 or 1 µg/mL, see **Figure 7C and 7D**, respectively). Ghrelin binding was also observed in the PVN; although positive cells were scarce (**Figure 7C–D**). Of note, the ghrelin binding signal in the PVN was weaker than the signal observed in the ARC region (compare inserts in A vs. inserts in D). To further assess GHSR gene expression in the ARC and PVN, we analyzed GHSR mRNA levels from micro-dissected punches of these brain nuclei. Similar to the relative GHSR levels determined with the ghrelin binding assay, the ARC demonstrated the highest GHSR mRNA levels (C_T_ = 24.7±0.3), whereas the PVN had significantly lower levels of GHSR mRNA (C_T_ = 28.4±0.4, P<0.01).

**Figure 8:**
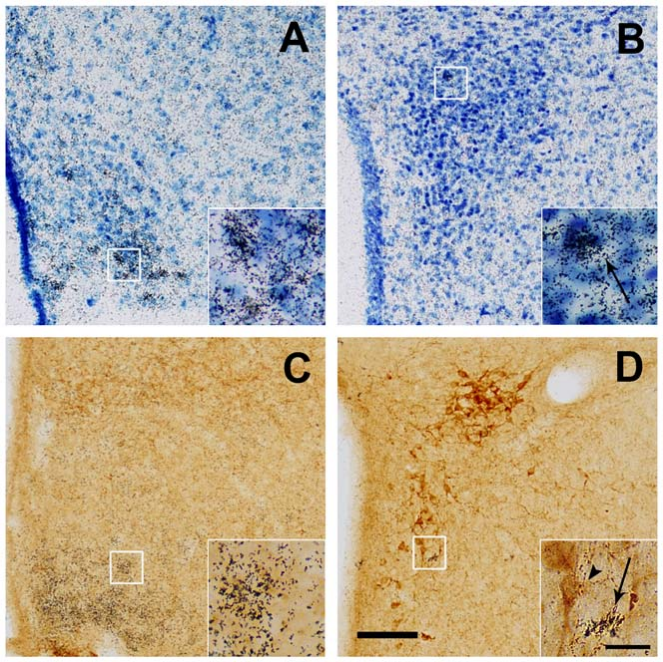
CRF-IR neurons of the PVN do not express GHSR. Upper panels show representative ISHH photomicrographs of thionin counterstained coronal sections of brain mouse. Panels **A** and **B** show photomicrographs of the ARC and PVN, respectively. Black silver granules, representing the binding of GHSR antisense riboprobes, can be detected at a higher density in the ARC (A) and with smaller density in the PVN (B). Bottom panels show representative photomicrographs of ISHH for GHSR (black silver granules) and IHC for CRF (brown staining) on coronal brain sections of mice. Panels **C** and **D** show photomicrographs of the ARC and PVN, respectively. Inserts in each image show high magnification of areas marked in low magnification images. White arrows point to examples of cells labeled with GHSR riboprobe. Black arrowhead point to examples of cells labeled with anti-CRF IHC. Scale bars, 100 µm (low magnification), 20 µm (high magnification).

To ascertain whether the GHSR-expressing neurons within the PVN included CRF neurons, we also performed ISHH for GHSR, as detailed in the past [32]. We confirmed previous results showing that the ARC contained abundant signal [32], which spanned the rostral-caudal expanse of the nucleus (**Figure 8A**). In comparison, the medial level of the PVN demonstrated few cells positive for GHSR mRNA expression (**Figure 8B**). In particular, less than 5% of the cells were positive for GHSR within the compact part of the mouse PVN, in our experimental conditions. To determine whether CRF neurons express GHSR, we performed ISHH for GHSR and IHC for CRF on the same coronal brain sections of mice. Again, we found abundant GHSR signal in the ARC (**Figure 8C**) and minimal GHSR signal in the compact part of the PVN. However, we were unable to detect cells positive for both CRF-IR and GHSR mRNA expression in the compact part of the PVN (**Figure 8D**).

## Discussion

The present study characterizes a role for ghrelin in signaling stress to the brain and activating the HPA neuroendocrine axis. Our results indicate that ghrelin is able to activate the CRF neurons of the PVN, and that this action is sufficient to acutely increase plasma glucocorticoid levels. However, the lack of detectable CRF and GHSR co-expressing neurons suggests that stimulation by ghrelin of the HPA axis occurs via an indirect mechanism. Interestingly, the ghrelin-induced activation of hypophysiotropic CRF neurons can occur independently of those circuitries controlling food intake. Thus, ghrelin's functions as an orexogenic signal and as a stress signal likely are dissociated anatomically.

The activation of the HPA neuroendocrine axis is a potential mechanism by which ghrelin regulates some physiological functions. For instance, it has been shown that the fat-accumulating effects of GHSR signaling are glucocorticoid dependent [33]. It is likely that the HPA axis also contributes to the mechanism by which ghrelin affects rewarding aspects of eating since glucocorticoids intensify motivated behaviors and increase intake of highly palatable foods [34]. The activation of the HPA axis may also be important when ghrelin plays a role defending against depressive symptoms of chronic stress. In this regard, we have shown that GHSR-null mice have intensified social isolation behavior and fail to have a full response of the HPA axis, as compared to wild type mice, in response to chronic social defeat stress [15]. Thus, it is likely that the ghrelin-induced activation of the HPA axis is one of the physiological mechanisms by which ghrelin helps animals to cope with stress.

It had previously been shown that ghrelin activates the HPA axis by acting at the hypothalamic level. Activation of GHSR signaling stimulates CRF mRNA expression in the hypothalamus when administrated *in vivo* and releases CRF from hypothalamic explants *in vitro* [35]. Moreover, central ghrelin infusion stimulates corticotrope cell hypertrophy and proliferation, and promotes the release of ACTH and corticosterone [19], [21]. Here, we show that ghrelin-responsive CRF neurons are concentrated in the compact part of the PVN, where most of hypophysiotropic neurons are located [29]. Ghrelin treatment also increases plasma corticosterone levels suggesting that ghrelin-induced activation of the HPA axis is likely due to increased secretion of CRF into the portal hypophysiotropic system. Interestingly, local action of ghrelin within the PVN is sufficient to activate the HPA axis. Future studies will be required to provide evidence of how and when ghrelin reaches the PVN. In mice, peripheral acyl-ghrelin crosses the blood brain barrier at a low rate [36], and its flow into the central nervous system varies among brain regions and serum factors [37]. It is currently unclear whether physiological fluctuations in plasma ghrelin can regulate the HPA axis. However, our previous finding that corticosterone levels are higher in chronically stressed wild-type mice as compared to chronically stressed GHSR-null mice suggests that an intact ghrelin signaling is required for the full activation of the HPA axis [15]. Although controversial, some evidence suggests that ghrelin is also produced in the brain and neuroanatomical studies have shown ghrelin-IR fibers innervating the PVN [38], [39]. Thus, a potential role of centrally-produced ghrelin as a modulator of CRF-producing neurons cannot be excluded.

The strong observed effect of ghrelin on the hypophysiotropic CRF neurons supports the concept that this hormone may play a role in the physiological regulation of the HPA axis activity. As indicated, previous studies have shown that plasma ghrelin rises in response to acute or chronic stress in both rodents and human beings [6]–[16]. The mechanisms responsible for the stress-associated increase in plasma ghrelin have not yet been determined; however, they likely depend on the sympathetic nervous system and/or release of catecholamines [40], [41]. Elevated plasma ghrelin could participate in the acute or chronic activation of the HPA axis in conditions such as upon fasting or caloric restriction, respectively. For instance, the HPA axis rapidly readjusts its set point under chronic stress, and a disruption of the glucocorticoid negative feedback system is observed [42], [43]. Thus, it can be hypothesized that the hypothalamic action of elevated ghrelin could override the negative feedback mediated by glucocorticoid. In the past, we have shown that leptin affects the hypophysiotropic TRH neurons sensitivity to the thyroid hormones and, as a consequence, the hypothalamic-pituitary-thyroid axis changes its set point to negative feedback mechanisms [44], [45]. Whether chronically elevated ghrelin levels would affect the activity and/or set point of the HPA axis will require further study.

The neuronal circuits mediating the ghrelin-induced activation of the HPA axis have not been thoroughly investigated. A direct action of ghrelin on the PVN neurons is relevant since it seems to be independent of the ghrelin-induced food intake. Of note, previous studies have reported that administration of a similar amount of ghrelin into the PVN of rats increases food intake [46]–[50]. The reason for this discrepancy with our data is unclear; although species differences might contribute to the difference. In fact, mouse and rat PVN have neuroanatomical differences [29] and differential GHSR expression between these species exists in the PVN [32]. The coronal level of the PVN where ghrelin acts could be another potential reason of the difference. In our experiments ghrelin was administrated specifically on the compact part of the PVN (Figure 5A), where most of hypophysiotropic CRF neurons are located [29]. Unfortunately, the above mentioned articles do not include photomicrographs or depictions of the injectors' tracts to establish this type of analysis. Also, Olszewski et al. showed an increase in c-fos-IR in the ARC in the intra-PVN injected animals, in contrast to our results, suggesting that a different neural circuit may be recruited in rats [46].

Here, we show that ghrelin indirectly regulates hypophysiotropic CRF neurons, as they failed to expresses GHSR mRNA or bind exogenous ghrelin in basal conditions. Thus, a number of potential indirect mechanisms should be considered. One possibility is that the ghrelin-induced activation of CRF neurons occurs indirectly via ghrelin action at a presynaptic level on neuronal terminals coming from the ARC [39]. The ARC plays a key role in the regulation of the energy balance [51]. It is known that GHSR is highly expressed in the Neuropeptide Y (NPY)-producing neurons of the ARC, and that ghrelin directly activates these neurons [52]–[54]. Some evidence suggests that the activation of NPY neurons is critical for ghrelin-induced increase of food intake [3], [55]. However, if NPY also mediates the ghrelin-induced activation of the CRF neurons is unknown. Such possibility is supported by previous reports showing that NPY neurons strongly innervate the CRF neurons of the PVN [56], CRF neurons express NPY receptors [57], and CRF neurons are directly activated NPY [58]. In addition, NPY inhibits inhibitory γ-aminobutyric acid inter-neurons of the PVN and thereby indirectly could increase the activity of the CRF neurons [39]. Of note, this potential mechanism for the ghrelin-induced activation of CRF neurons might be presynaptic and independent of the transcriptional activity in the GHSR-expressing neurons, as indicated by the finding that local intra-PVN injections of ghrelin induces activation of CRF neurons without any increase of c-fos in the ARC (Figure 5). As previously shown, we have confirmed that some PVN neurons express GHSR [32]. Thus, ghrelin action on non-CRF-containing PVN neurons that synapse onto CRF-neurons also might explain the ability of intra-PVN ghrelin administration to activate the HPA axis. Further studies will be needed to better establish the pathways mediating ghrelin's action on the hypophysiotropic CRF neurons.

These studies will help us better understand how the neuronal circuits that regulate eating behaviors are coordinated with some neuroendocrine responses key to maintain body homeostasis. The ghrelin-induced activation of the HPA axis is likely important acutely to reestablish the internal homeostasis, and also chronically to mediate some positive long-term effects of ghrelin on blood glucose and body weight regulation. It is hoped that these investigations will contribute for the future development of therapies to treat stress-associated alterations in energy balance, such as types of obesity or anorexia nervosa.
